# Shifted Coupling of EEG Driving Frequencies and fMRI Resting State Networks in Schizophrenia Spectrum Disorders

**DOI:** 10.1371/journal.pone.0076604

**Published:** 2013-10-04

**Authors:** Nadja Razavi, Kay Jann, Thomas Koenig, Mara Kottlow, Martinus Hauf, Werner Strik, Thomas Dierks

**Affiliations:** 1 Department of Psychiatric Neurophysiology, University Hospital of Psychiatry, University of Bern, Bern, Switzerland; 2 Ahmanson-Lovelace Brain Mapping Center, Department of Neurology, University of California Los Angeles, Los Angeles, California, United States of America; 3 University Institute of Diagnostic and Interventional Neuroradiology, Inselspital and University of Bern, Bern, Switzerland; Laureate Institute for Brain Research and The University of Oklahoma, United States of America

## Abstract

**Introduction:**

The cerebral resting state in schizophrenia is altered, as has been demonstrated separately by electroencephalography (EEG) and functional magnetic resonance imaging (fMRI) resting state networks (RSNs). Previous simultaneous EEG/fMRI findings in healthy controls suggest that a consistent spatiotemporal coupling between neural oscillations (EEG frequency correlates) and RSN activity is necessary to organize cognitive processes optimally. We hypothesized that this coupling is disorganized in schizophrenia and related psychotic disorders, in particular regarding higher cognitive RSNs such as the default-mode (DMN) and left-working-memory network (LWMN).

**Methods:**

Resting state was investigated in eleven patients with a schizophrenia spectrum disorder (n = 11) and matched healthy controls (n = 11) using simultaneous EEG/fMRI. The temporal association of each RSN to topographic spectral changes in the EEG was assessed by creating Covariance Maps. Group differences within, and group similarities across frequencies were estimated for the Covariance Maps.

**Results:**

The coupling of EEG frequency bands to the DMN and the LWMN respectively, displayed significant similarities that were shifted towards lower EEG frequencies in patients compared to healthy controls.

**Conclusions:**

By combining EEG and fMRI, each measuring different properties of the same pathophysiology, an aberrant relationship between EEG frequencies and altered RSNs was observed in patients. RSNs of patients were related to lower EEG frequencies, indicating functional alterations of the spatiotemporal coupling.

**Significance:**

The finding of a deviant and shifted coupling between RSNs and related EEG frequencies in patients with a schizophrenia spectrum disorder is significant, as it might indicate how failures in the processing of internal and external stimuli, as commonly seen during this symptomatology (i.e. thought disorders, hallucinations), arise.

## Introduction

Schizophrenia is a severe mental disorder, with a prevalence of 0.4–1% in the world population [1], [2]. The phenomenology of psychotic disorders in general is highly heterogeneous, challenging the identification of a single specific underlying biological mechanism. One generally discussed hypothesis of schizophrenia and related psychotic disorders is the “dysconnection syndrome” [3]–[6]. According to this hypothesis, the behavioral and cognitive deficits seen in patients with a schizophrenia spectrum disorder are caused by disturbed functional and/or anatomical communication between distant brain regions, which normally constitute a network.

Functional neural networks have been identified using functional magnetic resonance imaging (fMRI) during the awake resting state (resting state networks [RSNs]). RSNs provide information about the spontaneous intrinsic neurophysiological network architecture of cortical activity and are defined as temporal correlations of spontaneous low-frequency blood oxygen level-dependent (BOLD) signal fluctuations (<0.1 Hz) across widely separated brain regions [7]–[10]. Currently, about a dozen discrete RSNs have been identified, primarily in healthy controls [11], [12]. They can be modulated by factors such as task engagement, vigilance, neurophysiological/neuropsychiatric diseases, or age [13]–[16]. Conversely, the resting state of a subject has been shown to influence stimulus processing and task performance [17], [18].

Among these RSNs, two relate to functional states that are particularly affected in schizophrenia: the first is the default mode network (DMN), whose overall activity is increased when no task is being performed. It is suggested to be involved in self-referential mental activity and emotional processing [13]. Furthermore, there is evidence that the functional connectivity between the brain regions constituting the DMN is altered in schizophrenia spectrum disorders [19]–[21]. The abnormalities in the DMN in patients with psychosis were interpreted as a failure in the processing of internal cognition, which might be related to symptoms such as hallucinations or ego disturbances [22], . Moreover, a disturbed baseline is likely to interact with other, task-positive networks, possibly leading to rivalry between the psychological functions of the two networks, resulting, for example, in the executive dysfunctions occurring in the schizophrenia spectrum disorders [24]–[28]. The second is the left working memory network (LWMN), which is present during rest, but increases its activity along with task-related cognition [29]–[31], and has been shown to be important for symptoms related to language and memory processing (i.e., formal thought disorders) in psychosis [32]. These two RSNs involve frontal and/or temporal regions, which have been repeatedly found to be affected in functional activation, functional connectivity as well as structural neuroimaging studies on schizophrenia spectrum disorders, and are therefore of major interest for the present study (for review see: [33], [34]).

Until recently, the dysconnection hypothesis has been mainly addressed by structural and functional connectivity analyses between regions of interest (within and/or between RSN), as well as whole brain connectivity alterations (for structural connectivity: [35]–[38]; for functional connectivity: [14], [39]–[43]). Results of such studies have indicated dysfunctional connectivity in schizophrenia spectrum disorders. According to a review by Pettersson-Yeo et al. [44], the main trend in schizophrenia is a reduction of connectivity, however, the findings are not unequivocal [21].

In addition to the functional connectivity shown by fMRI, intact communication between individual brain regions of a specific RSN is established by neuronal synchronization at given frequencies in the sub-second range [30], [45]–[47]. Since the activity of such extended and synchronous neural oscillations is readily measurable with the electroencephalogram (EEG), combined recordings of fMRI and EEG during resting state conditions offer a unique possibility to quantify the binding mechanisms of the different nodes constituting a RSN. In particular, the advantage of combined multimodal measurement lies in the fact that MR-based methods cannot asses fast synchronized neuronal activity [45], [48]–[50], but EEG provides a high temporal resolution, and thus gives information regarding the frequency and amplitude (spectral power) of neural assemblies. EEG frequencies can be used to describe different states of consciousness, also depending on their topographical pattern: The alpha rhythm characterizes mental inactivity during wakefulness and is most prominent during eyes closed conditions. The beta rhythm is often detected during mental tasks. Theta is associated with maturational processes, to stages of drowsiness and sleep, as well as to memory functions and mental tasks. Delta waves are dominant during deep sleep or in pathological processes [51]–[53]. The signal of a resting state EEG is composed of a mixture of the frequency bands, where the power of each frequency band is continuously varying. Even if resting state-EEG and -fMRI both show retest reliability, a combined measurement is necessary, as the spontaneous temporal fluctuations of the resting state EEG frequencies has to correspond to identical time-periods of the spontaneous fMRI BOLD-signal fluctuations.

Various EEG studies investigated spectral frequency alterations in psychotic disorders. In the review of Boutros et al. [54] spectral frequency alterations are consistently reported for decreased alpha power, increased occurrence of slow rhythms (theta and delta waves, particularly over the frontal regions), and increased beta waves in schizophrenia [54]–[60].

Until now, no simultaneous EEG/fMRI study has investigated RSNs in patients with schizophrenia spectrum disorders. However, in healthy controls, simultaneous EEG/fMRI studies have demonstrated strong correlations between the temporal dynamics of the relative activity of different RSNs and the simultaneously recorded EEG oscillations in the major frequency bands, regardless of functional connectivity between the regions constituting the specific RSN [30], [61], [62]. In the following, we will refer to the association between EEG spectral changes in relation to RSNs activity as functional coupling. Specifically, a study by Jann et al. [30] replicated and extended a finding of Mantini et al. [62] where the relative activity in higher cognitive RSNs, i.e. the DMN, LWMN, or dorsal attention network (in comparison to the somatosensory RSNs such as the occipital visual or somato-motor network), were positively associated with relative power of fast frequency bands (alpha1 and 2, beta 1 to 3). The inverse association was true for the slow frequency bands. Furthermore, in contrast to the previous studies, which collapsed the frequency band information into one single value, Jann et al. [30] included the topographical information of the frequency bands given by the single electrodes. As such, not only the degree of the contribution of the frequency bands to a RSN was elaborated, but also the specific topographical signature/fingerprint at each frequency and for each of the RSNs separately. Together, these findings indicate that the functional coupling between EEG activity and fMRI RSNs underlies a specific topographical architecture, which may be important for the correct functioning and communication of cognitive processes.

As outlined above, there is reason to believe that the coupling of the RSNs dynamics and EEG power fluctuations is altered in patients with a schizophrenia spectrum disorder as compared to healthy controls. The aim of this study was to explore alterations in the coupling between EEG power fluctuations and RSN dynamics (DMN and LWMN) in patients with psychosis by using simultaneous resting state EEG/fMRI. This study bridges the gap between the large body of data on EEG spectral changes and alterations of fMRI networks observed in schizophrenia related psychosis, closely following the procedure introduced by Jann et al. [30]. Furthermore, the study provides insight into the alterations of non-task related neuronal activity, which might explain the underlying mechanisms of specific symptoms observed in schizophrenia [24], [63].

## Materials and Methods

### 2.1 Ethics statement

Procedures are in accordance with the Declaration of Helsinki and were approved by the local ethics committee (Kantonale Ethikkommission Bern no. 192-05). After a complete oral and written explanation of the aims and procedures of the study, patients were included in the study only if they had read the study description, could correctly summarize the study procedures, and did not have any open questions regarding the study. Additionally, the capacity of the patients to provide informed consent was evaluated and confirmed by their treating psychiatrists, who were independent of the present study. All patients were informed that participation is absolutely voluntary and that they could decline participation at any timepoint, without reasoning and without being disadvantaged in any other way in the medical treatment. All included patients and controls provided written consent prior to the beginning of the examination and received a copy of their signed consent. Participation was unpaid.

### 2.2 Participants

At the University Hospital of Psychiatry in Bern, Switzerland eleven right-handed inpatients with a psychotic disorder of the schizophrenia spectrum, were included. All patients suffered either from schizophrenia (F20.0–F20.3; 295.1–295.4/295.6; n = 7) or from an acute and transient psychotic disorder (F23.0–F23.2; 297.1/298.8; n = 4), as determined by ICD-10 and DSM-IV, respectively [1], [64]. Exclusion criteria were neurological or severe medical disorders, substance abuse other than nicotine, co-morbid psychiatric disorders, and any contradictions for MRI. All patients received psychopharmacological treatment: 10 of the 11 patients received atypical antipsychotics, while 5 of the 11 patients received typical antipsychotics (for chlorpromazine equivalents see Table 1). Nine patients were additionally treated with other psychoactive substances: antidepressants (n = 2), mood stabilizers (n = 6), tranquilizers (n = 5; diazepam equivalents: mean = 2.69, SD = 3.22), or anticholinergics (n = 1). Duration of illness and number of episodes were obtained from the case files and from information provided by the patient.

**Table 1:** Demographic characteristics and clinical variables of the healthy control and schizophrenia spectrum patient group.

	SZ		CG				
	(N = 11)		(N = 11)				
	N	%	N	%	?2^a^	df	p
**Gender (M/F)**	7/4	63.6	7/4	63.6	0.818	1	0.366
	**Mean**	**SD**	**Mean**	**SD**	**t** b	**df**	**p**
**Age (years)**	30.77	6.4	31.16	6.66	0.137	20	0.802
**Duration of illness (years)**	6.55	5.29	na	.	.	.	.
**Age of Onset**	24	6	na	.	.	.	.
**Number of Episodes**	3	1.61	na	.	.	.	.
**CPZE**	664.73	495.76	na	.	.	.	.
**DPZE**	2.69	3.22	na	.	.	.	.
**PANSS Total**	52.82	14.39	na	.	.	.	.
**PANSS Positive**	13.55	5.84	na	.	.	.	.
**PANSS Negative**	13.91	7.01	na	.	.	.	.

a Chi-square test: Asymptotic significance (two-tailed): p<0.05.

b independent samples *t*-test: significance (two-tailed): p<0.05.

SZ: Schizophrenia spectrum patient group.

CG: Healthy control group.

CPZE: chlorpromazine equivalents.

DPZE: diazepam equivalents.

PANSS: Positive and Negative Syndrome Scale.

SD: standard deviation.

df: degree of freedom.

The eleven age- and gender-matched healthy control subjects were partly drawn from a previous study [47]. Controls were included if they had no use of psychoactive medication, no consumption of illegal drugs, no history of neurological, severe medical, or psychiatric disorder, and fulfilled the criteria for MR measurement. Demographic characteristics for both groups, along with the clinical variables for the patients, are described in Table 1.

### 2.3 Data acquisition

Recordings of all subjects took place between 07.00 and 10.00 am to prevent possible circadian effects and control the intake of caffeine, alcohol or nicotine in the patient group.

#### 2.3.1 fMRI

All measurements were conducted on a 3T Siemens Magnetom Trio MR Scanner (Siemens, Erlangen, Germany). Firstly, functional T2*-weighted MR images were measured using an echo planar imaging sequence (TR/TE 1980 ms/30 ms, 32 slices, 252 volumes, 3×3×3 mm^3^, gap thickness 0.75 mm, matrix size 64×64, FOV 192×192 mm^2^). During the 9 min of simultaneous, functional measurement, participants were instructed to lie still, to relax with closed eyes, and to think of nothing in particular, without falling asleep (further information about movement control and statistical evaluation can be found in the Appendix S1). None of the subjects reported to have fallen asleep during the fMRI recordings. After simultaneous EEG-fMRI (see below), the EEG cap was removed, and T1-weighted anatomical data was recorded with a modified driven equilibrium Fourier transform [65] sequence (TR/TE 2300 ms/3.93 ms, 176 slices, slice thickness 1.0 mm, FOV 256×256 mm^2^).

#### 2.3.2 EEG

EEG data was acquired with an MR compatible EEG system from Brain Products (Gilching, Germany), with an input range of ±16.3 mV (16 bit resolution), and 92 channels. Electrodes were positioned in an elastic cap according to the international 10-10 system. Two additional electrodes were placed for electrocardiogram (below the clavicles) and 2 more for electrooculogram (below the eyes). The impedance of the electrodes was kept below 30 kΩ [66] and the measurement was conducted in an air-conditioned recording environment [67] (Appendix S1). Before the simultaneous acquisition, a reference EEG was recorded outside the scanner (6 min). The clock of the EEG system was synchronized with the clock of the MR system (10 kHz refresh rate) to facilitate artifact removal. The EEG was hardware bandpass filtered between 0.1 Hz and 250 Hz, digitally sampled, and stored at a rate of 5 kHz in order to minimize timing errors in the MR artifact removal [68].

### 2.4 Data preprocessing, extraction, and analysis

For an overview of the different analysis steps described below see Figure S1.

#### 2.4.1 fMRI

The processing of MR data was performed using the Brain VoyagerQX (Version 2.0.8.1480; Brain Innovation, Maastricht, The Netherlands). Preprocessing of the functional data included slice scan time correction, linear trend removal, 3D motion correction, and spatial smoothing with a Gaussian Kernel (FWHM 8 mm). Functional images were co-registered with the anatomical images, oriented to the anterior-posterior commissural plane, and normalized into standard Talairach space [69].

RSNs were identified by using a two-stage independent component analysis (ICA), which extracted 30 statistically maximal independent components (ICs) from the fMRI data. This method has already been used in previous studies [70], [71]. A spatial map and its mean dynamics across all voxels of the network were generated for each IC. Esposito and Goebel [72] demonstrated that the ‘best-fit’ DMN was found reliably in all of his investigated subjects, when a number between 30 to 50 ICA components was extracted. Hence, 30 ICs were extracted, and 2 raters (KJ and NR) assigned for each subject the DMN and LWMN, according to their spatial similarity and characteristic physiological fingerprints [11], [12], [73]–[75]. Possible differences in the assignment of the RSNs between the two raters were discussed until a mutual consent was found.

**Figure 1:**
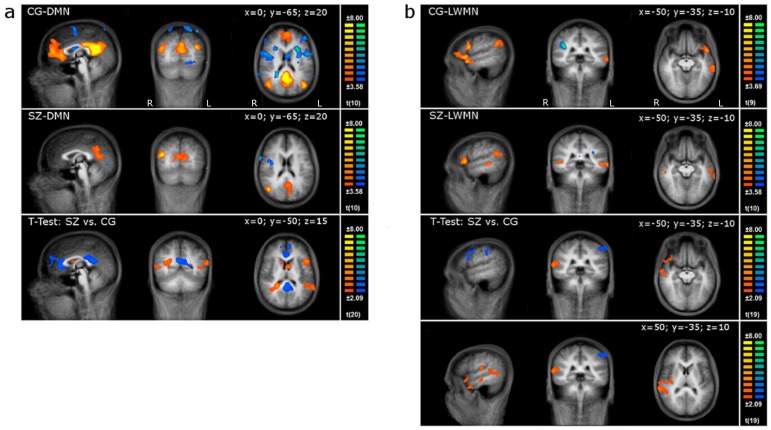
RSNs of healthy controls and patients. The first 2 rows depict the sagittal, coronal, and horizontal slices of the DMN (1a) and LWMN (1b) in the control (CG) and patient group (SZ; p<0.005; corrected at α<0.05; x, y, & z Talairach coordinates are given at the top right corner of each RSN, T-values are indicated at the right side of each analysis). The third and fourth lines display the *t*-test results between patients and controls (p<0.05, corrected at α<0.05; x, y, & z coordinates are given at the top right corner of each RSN, for the LWMN two additional slices are presented; T-values are indicated at the right side of each analysis). L: left; R: right.

Using a random effects *t*-test across the controls', as well as the patients' individual spatial DMN- and LWMN-ICs respectively, 2 group components (t-maps) were calculated that will be referred to as the CG (control group) and the SZ (schizophrenia spectrum disorder group) templates, respectively (Figure 1). For display, the threshold was set at p<0.005, and data was corrected for false positives with a spatial extent threshold at alpha 0.05 (Cluster Size Threshold Estimation using Monte Carlo simulations). Further, we computed the spatial similarity [30], [70] of each subject's IC to the CG-templates and tabulated the mean similarities (see Table 2, and for spatial similarity of the patients to their own SZ-template, see Table S1). The difference between the 2 group components was calculated using an unpaired *t*-test for each RSN (see Figure 1).

**Table 2:** The regions constituting the resting state networks of the control group component and the mean spatial similarity of each subject's independent component to the control group component.

RSN	x	y	z	Hemisphere	Anatomical Area	BA	mSS of CG	Assigned ICs of CG	mSS of SZ	Assigned ICs of SZ
**Default Mode Network (DMN)**	42.8	−66.6	20.6	Right	Inferior parietal lobe, junction of parietal temporal and occipital lobe	39	0.57	11	0.3079	11
	−0.4	44	12.5	Left	Dorsal anterior cingulate cortex	32				
	−1.8	−52.5	18.8	Left	Ventral posterior cingulate cortex	23				
	−40.4	−70.2	24.2	Left	Inferior Parietal Lobe, Junction of parietal temporal and occipital lobe	39				
**Left Working Memory or Language Network (LWMN)**	−19.1	48.7	36.2	Left	Superior frontal gyrus, DLPFC	9	0.42	10	0.2362	11
	−25.1	53.1	6.7	Left	Superior frontal gyrus, anterior prefrontal cortex	10				
	−43.3	9	33.7	Left	Middle frontal gyrus, DLPFC	9				
	−48	22.1	−3.8	Left	Inferior frontal gyrus, Orbital part	47				
	−49.8	−60.7	29.6	Left	Superior temporal gyrus, angular area/part of Wernicke's area	39				
	−61.6	−30.8	−9.6	Left	Middle temporal gyrus	21				

For each resting state network (RSN) the anatomical regions (BA: Brodmann area) included in the healthy control group (CG)-RSNs are listed. Anatomical areas are reported according to the Center of Gravity (Talairach Coordinates) in the group component. Mean spatial similarity (mSS) of each subject's independent component (IC) to the CG-group component is presented, in addition to the number of subjects assigned for each of the 2 RSNs. For mSS of the schizophrenia spectrum patients (SZ), ICs to their own SZ-template see Table S1. DLPFC: dorsolateral prefrontal cortex; DMN: default mode network; and LWMN: left working memory network.

Regarding the computation of the Covariance Maps, the temporal dynamics of the RSN in the individual data were extracted, using in-house generated MATLAB scripts as explained in the following. The individual, z-transformed BOLD dynamics of all 22 individual subjects were therefore weighted with the spatial CG-templates [30], which allowed us to extract the subject-specific relative signal fluctuation associated with each RSN as a function of time. The resulting time-courses were normalized for unit signal fluctuations across RSNs for each fMRI volume. By employing the CG-template for all subjects, we avoided the potentially confounding effects of individual or group differences in RSN spatial patterns. Furthermore, our main goal was to delineate the differences of patients' resting state processing in comparison to healthy controls'. However, since the results depend not only on the BOLD dynamics, but also on the fit of the individuals RSN pattern with the chosen template, we repeated the analysis using the SZ-template.

#### 2.4.2 EEG

EEG preprocessing was performed using Vision Analyzer (Version 1.05.0005; Brain Products, Gilching, Germany). EEG data was corrected for artifacts, including scan-pulse artifact correction, using average artifact subtraction [76]. Subsequently, data was bandpass filtered (1–30 Hz; Appendix S1). Additionally, remaining cardio-ballistic artifacts were corrected using a concatenated ICA of the outside and inside the scanner measured EEGs [47]. As such, the concatenated EEG allowed for a clear differentiation of scanner related artifacts: An increase in spectral power in the inside compared to the outside scanner recorded EEG, indicated scanner environment related ICs [47], [77]. EEG periods with residual scanner or movement artifacts were marked by visual inspection and excluded from all further analyses. Finally, data was down-sampled to 100 Hz and recalculated to average reference.

The preprocessed EEG was segmented into an equal number of epochs as MR volumes. An EEG epoch contained 256 data points, in a time window of −6560 ms to −4010 ms before each start of an MR volume [30]. This time window was chosen to meet the typical delay of 4–6 seconds in the hemodynamic response [78]. Thereafter, the segments were transformed using fast Fourier transformation (resolution 0.390625 Hz, Hanning window 10%).

For the calculation of the later explained combined EEG/fMRI analysis the segments were additionally averaged for the following frequency bands: 1.0< delta ≤3.5 Hz; 3.5< theta1 ≤6.25 Hz; 6.25< theta2≤8.2 Hz; 8.2< alpha1≤10.5 Hz; 10.5< alpha2≤14.0 Hz; 14.0< beta1≤18.75 Hz; 18.75< beta2≤21.88 Hz; 21.88< beta3≤30.0 Hz. The frequency band separation corresponds to the frequency bands evaluated by a cluster analysis of topographical Covariance Maps of the healthy control group in a previous study [30].

To validate our EEG only data with reference to earlier work on spectral EEG signatures in schizophrenia, we computed frequency bin wise group differences in the frequency spectra for the inside and outside the scanner recordings separately (Figure S2a (inside) and S2b (outside)). Individual global spectral power was calculated as the root mean square across all channels and averaged across all segments. Patients and controls were compared with frequency bin wise *t*-tests (Figure S2a and S2b). Based on the existing literature, lower alpha and increased theta and beta power was expected [54], [57]. These hypotheses were tested frequency bin wise using one-tailed t-tests.

### 2.5 Combining EEG and fMRI: Covariance Mapping

**Figure 2:**
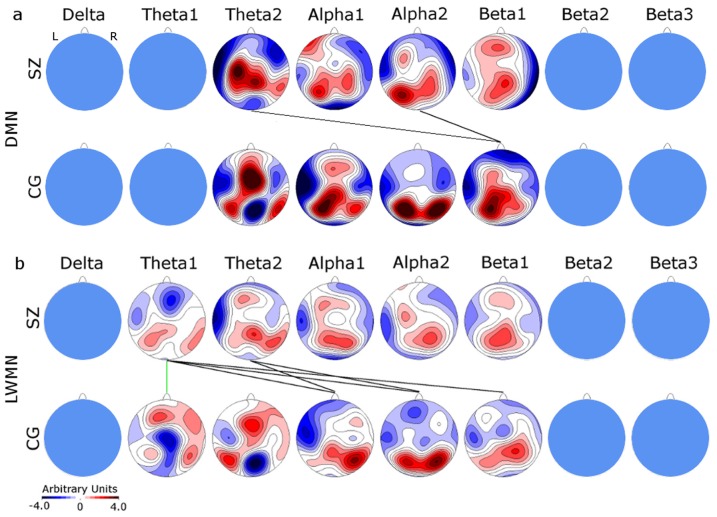
Topographical CovMaps for the DMN (2a) and LWMN (2b) at each of the 8 frequencies. The upper row displays the patients' (SZ) and the lower row the controls' (CG) CovMaps. Inconsistent CovMaps are blanked out. A positive covariance value (red) at a specific electrode indicates that the relative spectral power increased with the relative increase in the RSNs activity or isochronous decrease. Negative covariance values (blue) indicate decreased power when the RSN activity increased and vice versa. TANOVA and cross-correlation results, which were significant at α<0.05, are indicated by green, respectively black lines between the patients' and the controls' CovMap. L: left; R: right.

For the computation of the Covariance Maps the mean spectral amplitude across the epochs was removed from each channel and frequency band in the EEG, and the data for each epoch and frequency band had to be normalized to have unit variance across channels [30]. To calculate the topographic coupling between EEG and RSN (Covariance Maps), we dot-multiplied the individual dynamics of the normalized EEG spectral amplitudes at each electrode and frequency band with the normalized individual dynamics of the two RSNs. This was done for each subject separately resulting in a data matrix of 11 (subjects) ×2 (RSNs) ×8 (frequency bands) ×92 (electrodes) covariance values [30], [79]. Thereafter, the mean Covariance Map (CovMaps) for the patient and control groups were computed. Finally, the mean CovMaps were tested for consistency across subjects using the topographic consistency test [80], [81]. Using randomization procedures, this test estimates the probability of whether a mean topographic map has resulted from individual maps that have no common features. Only those CovMaps that were consistent for both groups were analyzed further (Figure 2, Figure S4).

#### 2.5.1 Between-groups analysis

*Topographical analysis of variance within frequency differences*: To calculate the topographical difference of the CovMaps between the groups and within one frequency band and RSN, topographical analysis of variance (TANOVA) was applied [81], [82]. TANOVA uses global indices for the estimation of map differences, as well as randomization procedures, in order to assess the significance of topographical differences. Using TANOVAs avoids having to a-priori select specific electrodes or having to correct for multiple testing across the electrodes. Where a TANOVA was significant, t-maps of the CovMap difference were computed to display the distribution of the difference (see Figure S3).

*Cross-correlation across frequency similarities*: In order to identify potential frequency shifts in the EEG-RSN signatures, we computed a correlation matrix among the CovMaps of the patient and control groups across all frequency bands. Because the resulting correlation coefficients were not computed from independent measurements, the significance of the correlation was established using the following randomization test. First, the correlation coefficient for the true similarity between patient- and control-mean CovMap for each RSN was calculated. Next, the group assignment of the individual CovMaps maps was randomly shuffled, 2 new group-mean CovMaps were computed for these randomly obtained groups, and the correlation coefficient between these 2 maps was calculated. This correlation coefficient was by definition, obtained under the assumption of the null hypothesis. By repeating the random assignment 5000 times, we obtained the random distribution of the correlation coefficient. The percentage of the 5000 randomly generated correlation coefficients that were larger than the true correlation coefficient was the probability (significance) that the similarity between the CovMaps of patients and controls has been obtained by chance.

#### 2.5.2 Within-group analysis

As all patients were under psychoactive medication, a TANCOVA was computed for all the CovMaps of the patients with the chlorpromazine equivalents as a continuous covariate [79]. The TANCOVA tests for the existence of a topographical map that is present in the individual data in a linear relationship to a continuous covariate. A significant result would indicate that the medication had a systematic influence on the topography of the CovMaps.

## Results

### 3.1 fMRI

The obtained group templates closely resemble previously reported RSNs (see Figure 1) [30], [73]. The DMN was identified for all controls and patients, while the LWMN was detected in 10 controls and 11 patients. The mean spatial similarity to the CG-template for both groups was above 0.2, similar to that found in previous studies (Table 2; and for SZ-Template: Table S1) [30], [70].

Compared to controls, the patients DMN showed decreased functional connectivity within the anterior cingulate cortex and posterior cingulate cortex, whereas within the right parahippocampal, left middle temporal, and left precentral regions, the opposite was true (Figure 1a and Table S2). This aberrant functional connectivity within the core regions of the DMN is in line with earlier reports [35], [83]. In the LWMN, compared to controls, the patients' functional connectivity was increased in the right superior and middle temporal gyrus, while the patients showed a widespread decreased functional connectivity in the bilateral middle frontal regions and left inferior parietal gyrus (Figure 1b and Table S2). These results are consistent with the existing literature, where compared to controls, patients exhibited increased functional connectivity in bilateral temporal regions [42].

### 3.2 EEG only

Related to the preprocessing of the EEG in the concatenated ICA there was no significant difference in the number of removed components between the groups (mean (± SD); CG: 22.2 (±3.9); SZ: 23.0 (±3.5); t = −0.066, df = 20, p = 0.95). Number of segments of the inside EEG recording for each group was sufficiently high for a subsequent analysis (mean (± SD); CG: 229.8 (±15.8); SZ: 206.9 (±14.9).

*EEG inside the scanner*: Frequency bin wise comparison of the spectra between the groups confirmed the expected lower power in the alpha1 & 2 range (9.8–11.3 Hz, t<−1.7247, df = 20, p = 0.05, one-sided; see Figure S2a). Moreover, an increase in the beta1 power (16.0 Hz, t>1.7247, df = 20, p = 0.05, one-sided) for the patients was observed, although this was at the exact frequency of the MR-scan pulse frequency, and was therefore discarded as an artifact. Additionally, our results showed a tendency toward increased delta and theta in patients (see Figure S2a).

*EEG outside the scanner*: In general, the peaks of the inside recordings coincide with the outside recordings (and vice versa), except for the peak in beta1, supporting the assumption of the MR-scan pulse artifact. However, the alpha peak of the outside recording did reach significance only by a trend, while at theta2 patients exhibited a significant increase (6.3–7.0 Hz, t>1.7247, df = 20, p = 0.05, one-sided, see Figure S2b).

### 3.3 Covariance mapping

The EEG Covariance Maps corresponding to the DMN and LWMN were mostly consistent (p<0.05) across controls, except for delta and beta3 in the DMN. Patients showed less consistent results: the topographic consistency test was negative in the DMN for delta, theta1, beta2, and beta3, and in the LWMN for delta, beta2, and beta3 frequencies. Further analysis included only CovMaps that were consistent for both groups (DMN: theta2, alpha1 & 2, beta1; LWMN: theta1 & 2, alpha1 & 2, beta1) (Figure 2). Results for the SZ-template were similar to those obtained using the CG-template (Figure S4).

In general, the CovMaps reflected the typical topographical distribution seen in the EEG of healthy subjects. Particularly, the relative spectral power increases in the alpha and beta frequencies seen in the posterior regions were associated with the relative increase in the RSNs activity, or isochronous decreases (red color in the CovMap) in the case of the control group (blue color in the CovMaps indicates inverse relation between spectral power and RSNs activity). However, in patients, this pattern was also observed in the theta frequencies. Furthermore, while controls displayed a left lateralized suppression of the alpha power during activity of the LWMN in anterior-temporal areas, patients expressed the same pattern additionally in the theta2 frequency, while their alpha CovMap demonstrated a more bilateral suppression. This is in accordance with the hypothesis of reduced lateralization in schizophrenia, where it is stated that a poor suppression of right-hemispheric activity may cause interference in processes such as language processing [42], [84]–[86].

#### 3.3.1 Between-group analysis

*Topographical group differences within frequencies*: The only significant group difference of Covariance Maps as assessed by a TANOVA was found in the theta1-band associated with the LWMN (p = 0.008; Figure 2b). The corresponding t-map indicated more LWMN associated theta-power in patients at central electrodes (t-max = 3.611 at electrode CP2) and less power at frontal electrodes (t-min = −3.038 at electrode AFz, Figure S3).

*Topographical group similarities across frequencies*: For the DMN, significant similarities were found between the patients' CovMaps of theta2 and alpha2 and the controls CovMap of beta1 (θ2-β1: r = 0.564, p = 0.005; α2-β1: r = 0.694, p = 0.027) (Figure 2a). In the LWMN, the patients' theta1 and 2 CovMaps were similar to the controls alpha1 (θ1-α1: r = 0.456, p = 0.023; θ2-α1: r = 0.605, p = 0.011) and 2 (θ1-α2: r = 0.401, p = 0.022; θ2-α2: r = 0.384, p = 0.036) maps; furthermore, the patients' theta1 CovMap resembled the beta1 (θ1-β1: r = 0.52, p = 0.013) map of the controls (Figure 2b).

Overall, we observed that similar maps were always associated with lower frequencies in the patients than in the controls. In addition, there was a “mixing,” in the sense that patients' and controls' maps did not follow a one-to-one association.

#### 3.3.2 Within-Group analysis

The TANCOVA did not reveal any significant effect of the antipsychotic medication on the CovMaps of the DMN and LWMN (p values were always above 0.26).

## Discussion

Our data demonstrates an altered coupling between RSNs (DMN and LWMN) and their driving oscillatory EEG frequencies in patients with a schizophrenia spectrum disorder. Interestingly, some CovMaps of the patients at lower frequencies best resembled those of controls at higher frequencies. These results are in line with previous resting state EEG investigations reporting a significant decrease in the alpha amplitude, and increases in delta, theta, and beta power in patients with a schizophrenia spectrum disorder [54], [56], [57]. In the following, we will discuss such an aberrant coupling in the context of its effects on cognitive functioning and putative psychopathological counterpart (including formal thought disorders, hallucinations, or executive dysfunctions).

In the DMN-CovMap, a similarity was observed between theta1 and alpha2 of patients and beta1 of controls. Some earlier resting EEG studies demonstrated that low frequency EEG alterations in patients tended to be localized to the frontal and posterior regions [55], [87]. However, due to the relatively limited spatial information provided by EEG, complex computational models are needed to estimate whether frequency power alterations match the regions constituting an RSN. Furthermore, the results of such models remain prone to the so called inverse problem [88]. More support for spatial network disturbances in schizophrenia spectrum disorders comes from resting state fMRI investigations showing alterations of the DMN in patients [20], [43].

For the LWMN, a shift from higher to lower frequencies was found for CovMaps of alpha1 & 2 in controls, and theta1 & 2 in patients, as well as for the association between beta1 in controls and theta1 in patients. These findings are in line with EEG reports on reduced lateralization effects in frontotemporal networks in schizophrenia, especially in the alpha band [89], [90]. The reduced lateralization of frontotemporal networks, similar but not identical to our LWMN, has also been demonstrated in various fMRI-task activation studies [42], [84]. In addition, general changes in the working memory networks of patients with schizophrenia have been demonstrated [91], [92].

Notably, the influence of the medication was statistically controlled for, and the results did not suggest any significant medication effects on the CovMaps of the patients.

Generally, the finding of an altered coupling between the EEG frequency bands and the DMN and LWMN may help to understand how internally triggered fundamental processing errors can occur in psychosis of schizophrenia spectrum disorders. The following four factors may be responsible for such an alteration in a uni- or multi-factorial way: dysfunctional cross-frequency binding to the RSNs, task-related suppression, vigilance, and maturational changes.

Firstly, the aberrant association could suggest that a clear coupling of specific frequencies to RSNs is required for proper cognitive processing. It has been proposed that cognitive RSNs such as the LWMN and DMN are primarily coupled to higher frequencies in the EEG [30]. In patients, however, the theta frequency showed associations with the LWMN and DMN, similar to the alpha and beta frequencies in controls. Therefore, it could be hypothesized that the aberrant association of frequencies and RSNs seen in patients enhances the risk of misinterpretation of inner processing by inducing noise. The noise could be described as dysfunctional cross-frequency binding to the RSNs. In healthy controls, the alpha-beta cross-frequency synchronization may actually represent different but coordinated attentional processes [93], which are functionally meaningful in their binding to higher-cognitive RSNs. Accordingly, the noisy coupling might disturb a functional self attentional process, by poor suppression or by false triggering of state-relevant networks. The finding of cross-frequency associations with RSNs is very striking in the investigation of neurophysiological alterations in schizophrenia spectrum disorders, as neither EEG nor fMRI alone were able to detect such an interplay between RSNs and multiple frequencies.

Secondly, the origin of the altered coupling in patients may partly be explained by results of various task-activation studies.

Several observations from fMRI studies in healthy subjects have shown that tasks requiring error monitoring and response inhibition engage brain regions that are part of the LWMN and DMN [91], [94]–[96]. Moreover, these tasks have been found to increase event-related low-frequency oscillations in the EEGs of healthy subjects (delta and theta), along with an alpha suppression [97], [98]. It was stated that the absolute power of the alpha and theta frequency bands, measured during a resting state, might predict memory performance, where individuals with more power in alpha and less power in theta perform better [98]. Moreover, a negative relationship between event-related oscillatory activity increases in low frequency and the spontaneous, resting oscillations is assumed [97], [98]. Supporting information for this hypothesis comes from a resting state study, where increased frontal theta power was found to be an index for decreased DMN activity using combined EEG/fMRI in healthy subjects and vice versa [99]. In contrast to healthy controls, schizophrenia patients exhibited an increased low-frequency power and a decreased alpha power at rest, while the expected increase of the low-frequency oscillations during task processing was reduced [54], [55], [100]. Hence, the present results indicate an altered recruitment of the DMN and LWMN (which are partly task relevant) in patients, by increased low-frequency oscillations, and in the case of the DMN a decreased alpha power, at rest. The over-engagement of task-relevant regions, at a task-relevant frequency, in networks that are active during an actual state of rest might present a physiological explanation for the occurrence of information processing errors in schizophrenia.

Thirdly, increased amplitude of the theta frequency, along with reduced alpha power, as observed in our study in relation to the DMN and LWMN in patients, has been found in wakefulness-sleep transitions or during vigilance modulations in healthy controls [101], [102]. Similar to our finding in patients, the hypnagogic state in the EEG exhibits a topographical shift of the alpha power from a posterior to a more anterior location [103]. A coupling between vigilance states (classified by EEG) and fMRI BOLD fluctuations was reported in control subjects [16]. These vigilance-related changes in brain activity showed overlapping regions with some RSNs associated with attention and vigilance shifts (e.g., DMN). Interestingly, reports of hypnagogic experiences in healthy subjects with reduced vigilance frequently include loss of control over and logic of thought [104], [105], phenomena similar to two of the major symptoms of psychotic disorders: hallucinations and formal thought disorder. Moreover, in a magnetoencephalography study, theta activity was reported to be increased over the left superior temporal cortex during spontaneous auditory hallucinations in a single patient with a schizophrenia disorder [90]. Therefore, one could argue that the dysfunctional recruitment of the DMN and LWMN by the theta frequency indicates a permanent abnormality in the regulation of vigilance. This, in relation to the self-monitoring and emotional processing functions of the DMN and the left-sided, language-related working memory functions of the LWMN, might explain the dysregulations and dysfunctional processing of internal and external stimuli in psychosis. Thus, it could be hypothesized that the shift of the CovMaps to a lower frequency indicates a sort of an open window, increasing the potential for hallucinatory events and/or incoherent processing of internal as well as external stimuli.

Finally, the dysfunctional coupling of spontaneous low-frequency EEG oscillations to the RSNs in schizophrenia spectrum disorders might be related to brain development. Most patients experience their first symptoms of schizophrenia between the ages of 15 and 25 [106]. Recently, maturational changes in brain structure and accompanying electrophysiological changes during adolescence were reviewed [107]; with increasing age, low-frequency EEG power reductions are accompanied by grey matter volume reductions. In schizophrenia, however, this brain maturation might be incomplete or deficient, indicating a neurodevelopmental origin [108], [109]. In healthy participants, transitions from slower to faster EEG frequencies from adolescence to adulthood but no differential coupling to the BOLD signal between the groups was reported at a late adolescent stage [15]; this may be because of an already mature pattern of EEG-BOLD coupling. However, the altered coupling observed in the present study might be the result of an incomplete neurodevelopmental process.

### Limitations and recommendations

There are a number of limitations to the current study. Firstly, there are many variables that contribute to the variance within our sample, such as the psychopathological profile, the duration of illness, and medication. However, this study did not aim to investigate the course of illness nor subgroups or symptom-specific changes in schizophrenia spectrum disorders. Regarding psychopharmacology we could replicate the alterations in the spectral pattern of the EEG that have previously been observed in in medicated, as well as medication naive patients [54], [55] (see Figure S2). Additionally, when the CPZE was controlled for in one of our statistical analyses, no medication effect was detectable (for a more detailed discussion of the psychopharmacological influence on the EEG see Appendix S1). Nevertheless, as in most of the studies with psychotic patients it cannot be completely excluded that medication has an influence. However, it seems questionable whether the employed experimental procedure would be feasible for untreated, acute psychotic patients. Simultaneous recordings are time consuming and very sensitive to subject compliance. A larger sample would open the possibility to analyze such variance inducing factors in the coupling to functionally relevant RSNs. There, subgroups of schizophrenia spectrum disorders, single-symptoms, duration of illness, or medication could be held constant. For the specificity of the results of schizophrenia spectrum patients, comparisons to other psychiatric disorders need to be conducted.

The selection of the DMN and LWMN template are based on unblinded human ratings of single subject ICAs rather than existing automated, group techniques. However, it has been demonstrated that automated techniques sometimes suffer specificity and attribute artifact components to a physiological DMN or LWMN, while the human interrater technique nearly achieved perfect correspondence to DMN [75] (for further information about selection techniques see Appendix S1: Blinded, unblinded and automated selection). In addition, the presently applied two-level approach (first subject-individual and then second-level group component computation) has been used in previous studies and demonstrated beneficial features, when systematic noise differences are to be expected between subjects or groups, which is the case in the slightly increased levels of motion in our patient sample [70], [72] (see Appendix S1: Movement control).

Finally, the computation of the relative covariance between the temporal fluctuations in the EEG and fMRI is limited to simultaneously occurring fluctuations. Delayed covariances could thus not be detected.

## Conclusion

Here, we demonstrate an altered coupling between functionally relevant RSNs and their driving neuronal oscillatory frequencies in patients with schizophrenia spectrum disorders. This investigation of abnormalities in this coupling will increase the understanding of the biological mechanisms of dysfunctional cognition and information processing commonly seen in schizophrenia related psychosis.

## Supporting Information

Figure S1 **Schematic representation showing the procedure of the statistical analysis conducted in this study.** The numbers indicate the section in the main text that describes the corresponding method in detail.
(PPTX)

Figure S2 **Spectral differences in the EEG of patients and controls.** Previous schizophrenia research has shown increased delta, theta, and beta amplitude, as well as decreased alpha power in patients with schizophrenia spectrum disorders (upper row, blue line) compared to controls (colored spectra). The lower row displays the results of t-test between the global spectral power (the root mean square across all channels and all segments) of the 2 groups. Dashed line: level of significance (t±1.7247, df = 20, p<0.05, one-sided). Figure S2a depicts the inside the scanner recordings, while Figure S2b shows the outside the scanner recordings.
(TIF)

Figure S3 **T-Maps showing the significant TANOVA results for the LWMN.** The CG-template was used at theta 1 (a: t-max = 3.611 at CP2; t-min = −3.038 at AFz), and the SZ-template at theta 1 (b: t-max = 3.728 at CPz; t-min = −2.779 at F5) and beta 1 (c: t-max = 4.632 at AFz; t-min = −2.652 at P10). All tests were computed using an unpaired t-test at p<0.05. L: left; R: right; T: T-values.
(TIF)

Figure S4 **Figures S4a and S4b show the topographic Covariance Maps (CovMaps) for the DMN and LWMN, respectively, for the 8 frequencies.** Here, for each group, their own template was used; i.e., patient (SZ) CovMaps was calculated with the SZ-template and the controls' (CG) with the CG-template. The upper row displays the patients' and the lower row the controls' CovMaps. Inconsistent CovMaps are blanked out. A positive covariance value (red) at a specific electrode indicates that the relative spectral power increased along with a relative increase in the RSNs activity or an isochronous decrease. Negative covariance values (blue) indicate a decrease in power when the RSN activity increased and vice versa. TANOVA and cross-correlation results which were significant at α<0.05 are indicated by green, respectively black lines between the patients' and the controls' CovMaps. L: left; R: right.
(TIF)

Table S1 The regions constituting the resting state networks of the patients group component and the mean spatial similarity of each patients' IC to the schizophrenia spectrum disorder group component.
(DOCX)

Table S2 Regions showing group differences in spatial maps of the DMN and LWMN.
(DOCX)

Appendix S1 **Additional information about influence of impedance on EEG data quality, effect of EEG bandpass filtering between 1–30 Hz, and limitations.**
(DOCX)
