# Supplementary figures and images for: Shifted Coupling of EEG Driving Frequencies and fMRI Resting State Networks in Schizophrenia Spectrum Disorders
Source: PLoS One. 2013 Oct 4;8(10):e76604. doi: 10.1371/journal.pone.0076604 (PMC3790692; doi:10.1371/journal.pone.0076604)

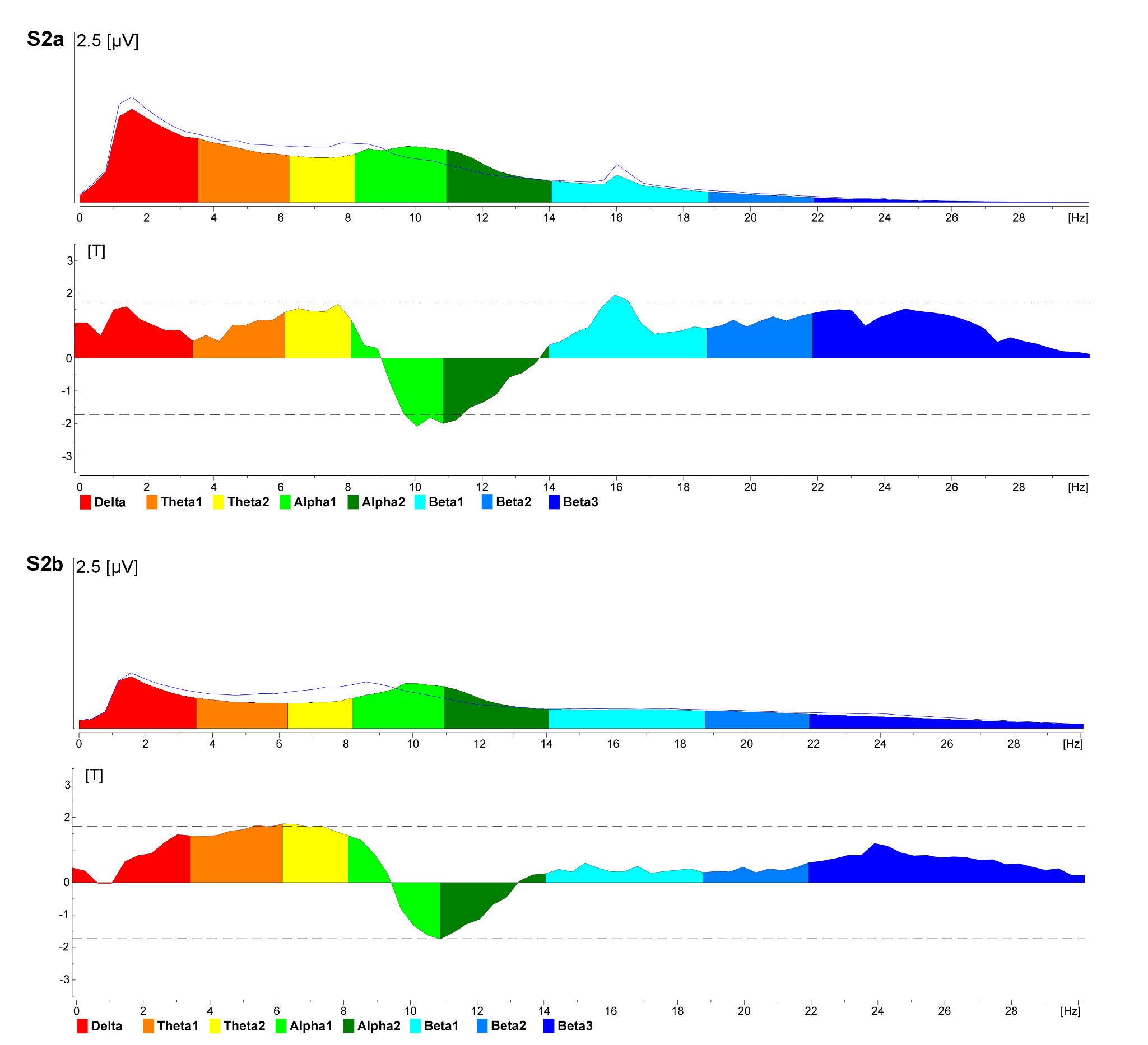

Supplement: Figure S2 — Spectral differences in the EEG of patients and controls. Previous schizophrenia research has shown increased delta, theta, and beta amplitude, as well as decreased alpha power in patients with schizophrenia spectrum disorders (upper row, blue line) compared to controls (colored spectra). The lower row displays the results of t-test between the global spectral power (the root mean square across all channels and all segments) of the 2 groups. Dashed line: level of significance (t±1.7247, df = 20, p<0.05, one-sided). Figure S2a depicts the inside the scanner recordings, while Figure S2b shows the outside the scanner recordings. (TIF) [file pone.0076604.s002.tif]

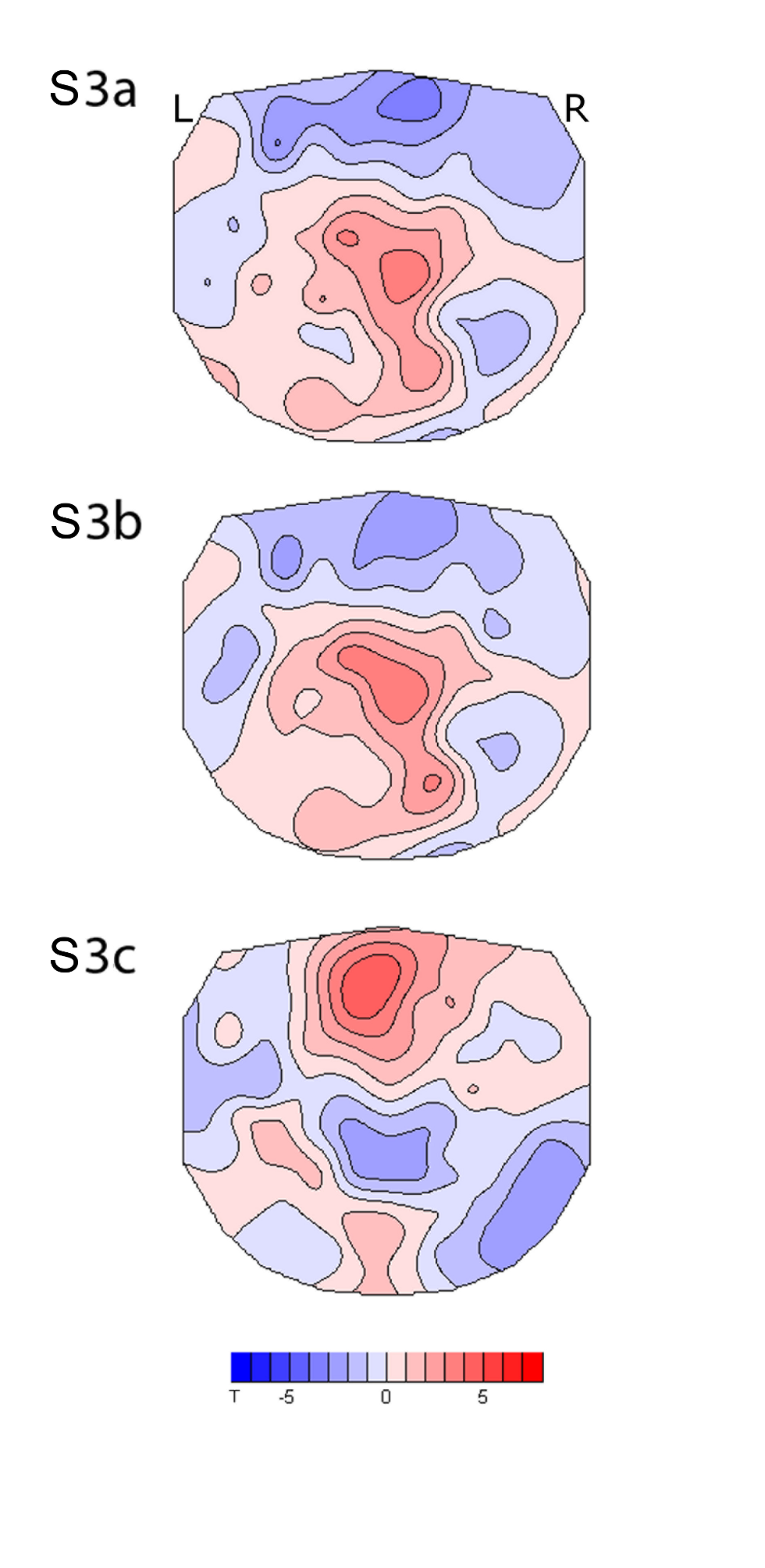

Supplement: Figure S3 — T-Maps showing the significant TANOVA results for the LWMN. The CG-template was used at theta 1 (a: t-max = 3.611 at CP2; t-min = −3.038 at AFz), and the SZ-template at theta 1 (b: t-max = 3.728 at CPz; t-min = −2.779 at F5) and beta 1 (c: t-max = 4.632 at AFz; t-min = −2.652 at P10). All tests were computed using an unpaired t-test at p<0.05. L: left; R: right; T: T-values. (TIF) [file pone.0076604.s003.tif]

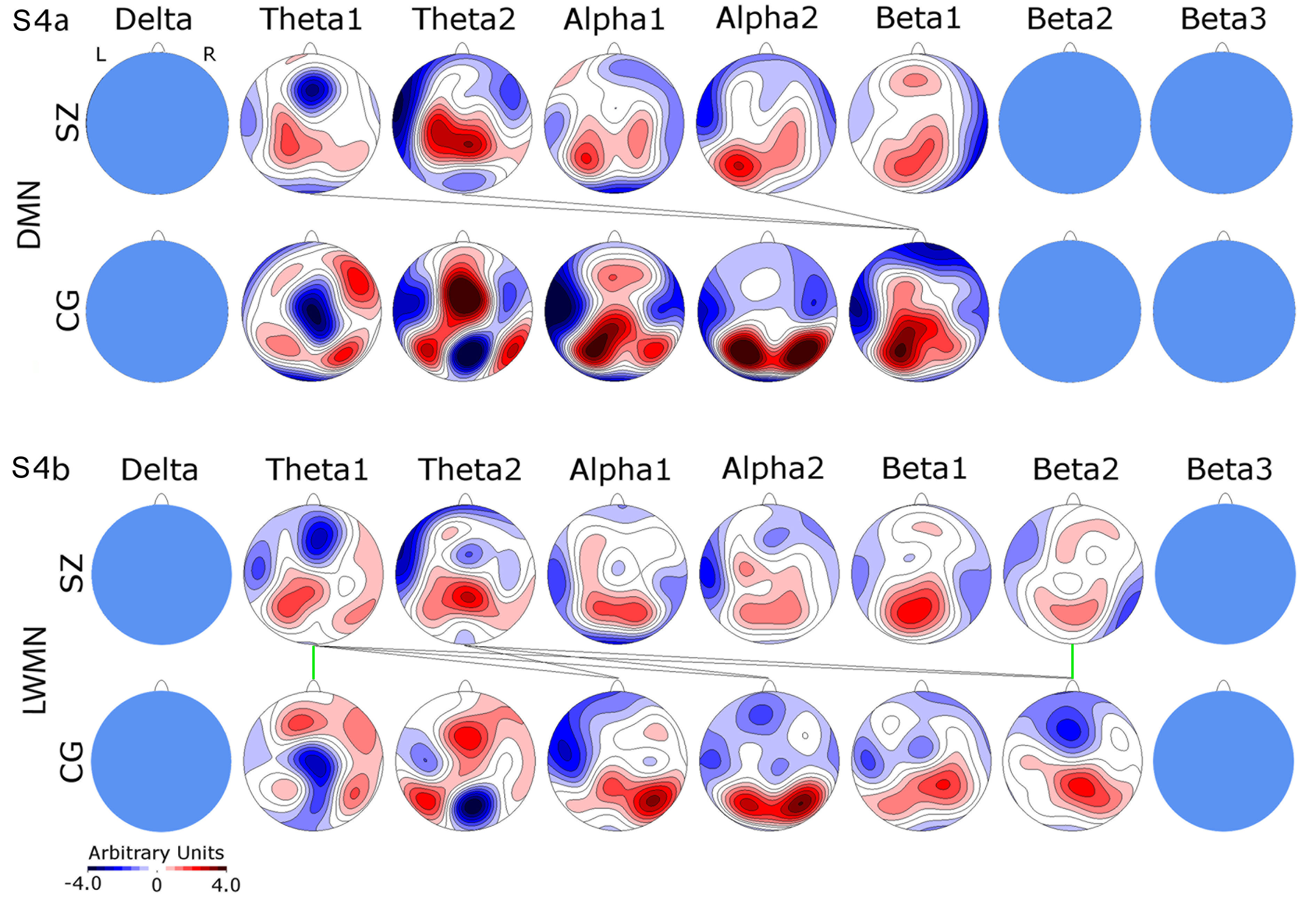

Supplement: Figure S4 — Figures S4a and S4b show the topographic Covariance Maps (CovMaps) for the DMN and LWMN, respectively, for the 8 frequencies. Here, for each group, their own template was used; i.e., patient (SZ) CovMaps was calculated with the SZ-template and the controls' (CG) with the CG-template. The upper row displays the patients' and the lower row the controls' CovMaps. Inconsistent CovMaps are blanked out. A positive covariance value (red) at a specific electrode indicates that the relative spectral power increased along with a relative increase in the RSNs activity or an isochronous decrease. Negative covariance values (blue) indicate a decrease in power when the RSN activity increased and vice versa. TANOVA and cross-correlation results which were significant at α<0.05 are indicated by green, respectively black lines between the patients' and the controls' CovMaps. L: left; R: right. (TIF) [file pone.0076604.s004.tif]
