# Supplementary material for: Shifted Coupling of EEG Driving Frequencies and fMRI Resting State Networks in Schizophrenia Spectrum Disorders
Source: PLoS One. 2013 Oct 4;8(10):e76604. doi: 10.1371/journal.pone.0076604 (PMC3790692; doi:10.1371/journal.pone.0076604)
Supplement: Table S1 — The regions constituting the resting state networks of the patients group component and the mean spatial similarity of each patients' IC to the schizophrenia spectrum disorder group component. (DOCX) [file pone.0076604.s005.docx]

**Supporting Tables**

**Table S1.** The regions constituting the resting state networks of the patients group component and the mean spatial similarity of each patients' IC

to the schizophrenia spectrum disorder group component.

| **RSN** | **x** | **y** | **z** | **Hemisphere** | **Anatomical Area** | **BA** | **mSS of SZ** | **Assigned ICs of SZ** |
| --- | --- | --- | --- | --- | --- | --- | --- | --- |
| **Default Mode Network (DMN)** | 45 | -65.3 | 17.6 | Right | Inferior parietal lobe, junction of parietal temporal and occipital lobe | 39 | 0.42 | 11 |
|  | -2.3 | -58.8 | 18.7 | Left | Ventral posterior cingulate cortex | 23 |  |  |
| **Left Working Memory or Language Network (LWMN)** | 50.4 | -35.3 | -1.3 | Right | Middle temporal gyrus | 21 | 0.41 | 11 |
|  | -47.5 | 22.8 | 2.9 | Left | Inferior frontal gyrus, orbital part | 47 |  |  |
|  | -55.8 | -58.1 | 14.7 | Left | Superior temporal gyrus, angular area/part of Wernicke's area | 22 |  |  |
|  | -57.8 | -30.7 | -5.6 | Left | Middle temporal gyrus | 21 |  |  |

^a^ For each resting state network (RSN) the anatomical regions (BA: Brodmann area) included in the patients group (SZ)-RSNs are listed.

Anatomical area is reported according to the center of gravity (Talairach Coordinates) in the group component. Mean spatial similarity (mSS) of

each patients' Independent Component (IC) to the SZ-group component is presented, as well as the number of subjects assigned for each of the

two RSNs. ACC: anterior cingulate cortex; PCC: posterior cingulate cortex; DLPFC: dorsolateral prefrontal cortex.
