# Supplementary material for: Shifted Coupling of EEG Driving Frequencies and fMRI Resting State Networks in Schizophrenia Spectrum Disorders
Source: PLoS One. 2013 Oct 4;8(10):e76604. doi: 10.1371/journal.pone.0076604 (PMC3790692; doi:10.1371/journal.pone.0076604)
Supplement: Table S2 — Regions showing group differences in spatial maps of the DMN and LWMN. (DOCX) [file pone.0076604.s006.docx]

**Supporting Tables**

**Table S2.** Regions showing group differences in spatial maps of the DMN and LWMN. These regions are displayed in Figure 1.

| **RSN** |  | **x** | **y** | **z** | **Hemisphere** | **Anatomical Area** | **BA** |
| --- | --- | --- | --- | --- | --- | --- | --- |
| **DMN** | Decreased functional connectivity | -2.94 | 43.02 | 13.07 | Left | ACC | 32 |
|  |  | -2.55 | -41.85 | 19.75 | Left | PCC | 29 |
|  | Increased functional connectivity | 38.02 | -40.87 | 1.41 | Right | Parahippocampal gyrus | 19 |
|  |  | -52.02 | -41.27 | 3.3 | Left | Middle temporal gyrus | 22 |
|  |  | -49.35 | 10.87 | 11.76 | Left | Precentral gyrus | 44 |
|  |  | 20.98 | -0.95 | 31.26 |  | Not within gray matter |  |
|  |  | -19.12 | -3.78 | 27.52 |  |  |  |
| **LWMN** | Decreased functional connectivity | 23.83 | 20.89 | 38.14 | Right | Middle frontal regions | 8 |
|  |  | 28.67 | 10.68 | 44.08 | Left | Middle frontal regions | 6 |
|  |  | -44.23 | -36.24 | 44.08 | Left | Inferior parietal gyrus | 40 |
|  | Increased functional connectivity | 54.98 | -33.89 | 13.05 | Right | Superior temporal gyrus | 42 |
|  |  | 54.48 | 5.52 | -18.18 | Right | Middle temporal gyrus | 21 |

^a^ RSN: resting state network; BA: Brodmann area; DMN: default mode network; LWMN: left working memory network; ACC: anterior cingulate cortex; PCC: posterior cingulate cortex; DLPFC: dorsolateral prefrontal cortex.
