# Supplementary material for: Shifted Coupling of EEG Driving Frequencies and fMRI Resting State Networks in Schizophrenia Spectrum Disorders
Source: PLoS One. 2013 Oct 4;8(10):e76604. doi: 10.1371/journal.pone.0076604 (PMC3790692; doi:10.1371/journal.pone.0076604)
Supplement: Appendix S1 — Additional information about influence of impedance on EEG data quality, effect of EEG bandpass filtering between 1–30 Hz, and limitations. (DOCX) [file pone.0076604.s007.docx]

**Supporting Information: Appendix S**

***Influence of impedance on EEG data quality:***

It may be argued that group differences of EEG power might arise from differences in electrode impedances. However, [Ferree et al. [1](#_ENREF_1)] demonstrated in their study that there is no significant amplitude change in any EEG frequency band, as impedance varied between 10 to 40 kΩ. Furthermore, modern high input-impedance amplifiers, accurate digital filters for power line noise, and a cool and dry recording environment as used in our study, reduce the possibility of substantial contribution of impedance artifacts in the data [[2](#_ENREF_2)]. Nevertheless, an analysis of the correlations between electrode impedances and EEG power indicated that such effects are, if present, nearly constant across frequencies, suggesting that electrodes with higher impedances are more affected by white noise. The clearly frequency specific group differences observed here are thus not compatible with mere impedance differences. Furthermore, possibly lower quality of the fMRI gradient artifact elimination procedure in the patients due to increased motion artifacts is expected to yield effects at the frequency of the MR gradients and its’ harmonics. In our settings, the MR gradients were at 16.6 Hz, which is clearly above the frequencies affected in the patients.

***Effects of EEG bandpass filtering between 1-30 Hz:***

EEG data was bandpass filtered in order to minimize artifacts related to environmental factors before ICA correction was used for the identification of cardio-ballistic and scan-pulse related artifacts [[3](#_ENREF_3),[4](#_ENREF_4)]. However, this filtering may lead to effects in the first and last analyzed bands, which do not allow for a reliable conclusion. Interpretations on these frequency bands therefore need to be done carefully. However, it must be kept in mind, that the Covariance Maps of the combined analysis were routinely checked for consistency. They were only included in the between and across frequency analysis if they were consistent for both groups. Since delta and beta 2 & 3 Covariance maps were not consistent, they were anyway excluded from any further combined EEG/fMRI analysis.

***Limitations:***

*Duration of illness:* The duration of illness in our sample included younger patients with a recent onset of psychosis (mostly classified as F23), as well as older patients with more chronic schizophrenia (F20). Apparently, chronicity affects data heterogeneity by moderating delta- and theta-frequency bands [[5](#_ENREF_5)]. However, this study did not aim to investigate the course of illness in schizophrenia spectrum disorders.

*Psychopharmacology:* As in most studies on psychotic disorders, medication may be a confounding factor in our study. Effects of psychotropic medication on EEG recordings have been reported previously. Generally, sedative agents cause a slowing of the EEG frequency, while highly potent typical antipsychotics increase the EEG frequency [[6](#_ENREF_6)]. Concerning atypical antipsychotics, clozapine induces a general increase of low-frequency amplitude and a decrease of high-frequency amplitude in the anterior regions of the brain [[7](#_ENREF_7)]. In the present study, only two patients, who had received a relatively low dose of clozapine were included. As a routine of our clinic all patients treated with clozapine have an EEG-monitoring. However, the reports of the independently acquired clinical EEG, did not suggest any obvious medication induced alterations. Further, the alterations seen in the EEG of psychopharmacologically treated patients were also observed in medication naïve and drug free patients and were even ameliorated under medication [[8](#_ENREF_8),[9](#_ENREF_9)]. Nonetheless, even though the CPZE was controlled for by one of the statistical analyses, a medication effect cannot be completely excluded.

*Blinded, unblinded, and automated selection of fMRI-ICs:* Regarding the comparison of fMRI-group templates, the unblinded determination of the RSNs might have biased this specific finding. In contrast to the fMRI-group statistics, a possible systematic bias in the selection of the RSNs in healthy controls or patients should not lead to a systematic bias in our Covariance Mapping. Since the same mean template of the healthy controls has been used to extract all CovMaps (patients and controls) the main finding of the downshift of the CovMaps in patients versus controls is unlikely to be affected by the way the components were selected (unblinded). We want to point out that for the computation of the CovMaps all voxels of all the individual, z-transformed BOLD dynamics were weighted with the spatial CG-templates (no threshold to the data).
However, in order to evaluate a possible selection bias that might affect the fMRI group statistics, we compared the spatial correlation coefficient of every single subject specific IC to a template DMN (derived from [[10](#_ENREF_10)]) using the inter-rater/method-reliability analysis “Cohen’s Kappa” for the following pairs: 1) unblinded-blinded; 2) unblinded-automated/mean spatial similarities; and 3) blinded-automated/mean spatial similarities. Cohen’s Kappa assesses the level of agreement between two methods on the assignment of independent components to the DMN or LWMN respectively. Results confirm that the reproducibility between the human-human reliability has an almost perfect agreement, while the human-automated reliability have a substantial agreement, thus replicating the results of [Franco et al. [11](#_ENREF_11)] and suggesting that the selection of the RSNs is not influenced by the blinding of the raters (KJ and NR, see **Table below)**.

| **Table:** Inter-method-reliability analysis “Cohen’s Kappa” for the subject-wise selection of RSN components. | | |
| --- | --- | --- |
|  |  | **Cohen's Kappa** |
| **DMN** | **unblinded vs blinded** | 0.80 |
|  | **unblinded vs mss** | 0.75 |
|  | **blinded vs mss** | 0.75 |
| **LWMN** | **unblinded vs blinded*** | 0.80 |
|  | **unblinded vs mss*** | 0.79 |
|  | **blinded vs mss** | 0.71 |
| *Only including 21 subjects since in the unblinded condition no LWMN was assigned. Mss: mean spatial similarity; p < 0.001. | | |

*Movement control:* According to our protocol, all participating subjects were instructed to lie still in the scanner. Moreover, subjects were excluded if they exhibited motions in the scanner above a value of 3mm or 3 degrees. In addition motion correction was performed on the datasets.
However, it has been shown, that systematic differences in motion between groups can affect the functional connectivity [[12](#_ENREF_12),[13](#_ENREF_13)] and that the conventional approach to correct for motion might not be sufficient to rule out a motion-bias in functional connectivity calculation.
To statistically compare the amount of motion in the scanner between the two groups, we computed the translational and rotational motion for each subject according to previous studies [[13](#_ENREF_13),[14](#_ENREF_14)]. We did not find any difference between the groups regarding the rotational movement (mean (±SD) CG: 0.37 (±0.24); SZ: 0.59 (±0.48); t = -1.46, df = 20; p = 0.16 (two-sided)). In the translational movement a significant group difference was detected (mean (±SD) CG: 0.05 (±0.01); SZ: 0.08 (±0.05); t = -2.56, df = 20; p = 0.03 (two-sided)).

Hence, there might be a systematic difference in functional connectivity due to motion in the two subject groups that affects the voxel-wise t-test of the networks between the two groups (Figure 1a & b in the manuscript). However, the main focus of the present work was on the relation between RSN dynamics and EEG spectral power fluctuations. Hence, the measure used for the Covariance Mapping was the weighted, relative, normalized signal fluctuations estimated from all voxels of the two networks of the Control Group only. Accordingly, differences in the strength of ROI-to-ROI functional connectivity or amplitude of the fluctuations do not bias the resulting RSN dynamic. Thus, our main results (CovMaps) are likely to be independent of local group differences in functional connectivity, as well as the strength of the connectivity.

Supplementary References

1. Ferree TC, Luu P, Russell GS, Tucker DM (2001) Scalp electrode impedance, infection risk, and EEG data quality. Clin Neurophysiol 112: 536-544.

2. Kappenman ES, Luck SJ (2010) The effects of electrode impedance on data quality and statistical significance in ERP recordings. Psychophysiology 47: 888-904.

3. Jann K, Dierks T, Boesch C, Kottlow M, Strik W, et al. (2009) BOLD correlates of EEG alpha phase-locking and the fMRI default mode network. Neuroimage 45: 903-916.

4. Kottlow M, Jann K, Dierks T, Koenig T (2012) Increased phase synchronization during continuous face integration measured simultaneously with EEG and fMRI. Clin Neurophysiol 123: 1536-1548.

5. Galderisi S, Mucci A, Volpe U, Boutros N (2009) Evidence-based medicine and electrophysiology in schizophrenia. Clin EEG Neurosci 40: 62-77.

6. Dierks T, Mueller T (2009) EEG and EP in Psychiatrc Disorders. Epileptologie 26: 21-27.

7. Lacroix D, Chaput Y, Rodriguez JP, Filion M, Morrison D, et al. (1995) Quantified EEG changes associated with a positive clinical response to clozapine in schizophrenia. Prog Neuropsychopharmacol Biol Psychiatry 19: 861-876.

8. Boutros NN, Arfken C, Galderisi S, Warrick J, Pratt G, et al. (2008) The status of spectral EEG abnormality as a diagnostic test for schizophrenia. Schizophr Res 99: 225-237.

9. Galderisi S, Mucci A, Mignone ML, Maj M, Kemali D (1991) CEEG mapping in drug-free schizophrenics. Differences from healthy subjects and changes induced by haloperidol treatment. Schizophr Res 6: 15-23.

10. Jann K, Kottlow M, Dierks T, Boesch C, Koenig T (2010) Topographic electrophysiological signatures of FMRI Resting State Networks. PLoS One 5: e12945.

11. Franco AR, Pritchard A, Calhoun VD, Mayer AR (2009) Interrater and intermethod reliability of default mode network selection. Hum Brain Mapp 30: 2293-2303.

12. Power JD, Barnes KA, Snyder AZ, Schlaggar BL, Petersen SE (2012) Spurious but systematic correlations in functional connectivity MRI networks arise from subject motion. Neuroimage 59: 2142-2154.

13. Van Dijk KR, Sabuncu MR, Buckner RL (2012) The influence of head motion on intrinsic functional connectivity MRI. Neuroimage 59: 431-438.

14. Patriat R, Molloy EK, Meier TB, Kirk GR, Nair VA, et al. (2013) The effect of resting condition on resting-state fMRI reliability and consistency: A comparison between resting with eyes open, closed, and fixated. Neuroimage 78: 463-473.
